# Demographic feedbacks during evolutionary rescue can slow or speed adaptive evolution

**DOI:** 10.1098/rspb.2023.1553

**Published:** 2024-02-14

**Authors:** Jeremy A. Draghi, Joel W. McGlothlin, Holly K. Kindsvater

**Affiliations:** ^1^ Department of Biological Sciences, Virginia Tech, Blacksburg, VA 24060, USA; ^2^ Department of Fish and Wildlife Conservation, Virginia Tech, Blacksburg, VA 24060, USA

**Keywords:** evolutionary rescue, demography, adaptation, evolutionary theory

## Abstract

Populations declining toward extinction can persist via genetic adaptation in a process called evolutionary rescue. Predicting evolutionary rescue has applications ranging from conservation biology to medicine, but requires understanding and integrating the multiple effects of a stressful environmental change on population processes. Here we derive a simple expression for how generation time, a key determinant of the rate of evolution, varies with population size during evolutionary rescue. Change in generation time is quantitatively predicted by comparing how intraspecific competition and the source of maladaptation each affect the rates of births and deaths in the population. Depending on the difference between two parameters quantifying these effects, the model predicts that populations may experience substantial changes in their rate of adaptation in both positive and negative directions, or adapt consistently despite severe stress. These predictions were then tested by comparison to the results of individual-based simulations of evolutionary rescue, which validated that the tolerable rate of environmental change varied considerably as described by analytical results. We discuss how these results inform efforts to understand wildlife disease and adaptation to climate change, evolution in managed populations and treatment resistance in pathogens.

## 1. Background

Evolutionary rescue occurs when a population declining toward extinction undergoes genetic adaptation that stabilizes population size [1,2]. Interest in evolutionary rescue is motivated by the recognition that evolution can often be rapid [3] and that many threatened species may depend on adaptation to persist in changing environments. The theory of evolutionary rescue also applies to predicting the fates of populations migrating to habitats to which they are initially poorly suited, with applications ranging from potentially invasive species [4] to pathogens spilling over into new hosts [5]. Preventing evolutionary rescue is also essential when treating evolvable threats such as bacterial infections and cancer [6].

The intuitive premise of evolutionary rescue is that stressful environments, in which reproductive success falls short of replacement, generally exert a selective pressure for improved fitness alongside their demographic effects [1]. However, eco-evolutionary feedbacks can add complications [7–9]. Theory on these feedbacks between maladaptation and the adaptive response has focused on three main elements: effective population size [10,11], heritability [12,13] and the strength of natural selection [14]. These reflect fundamental tenets of evolutionary and conservation biology: maladaptation is typically thought to decrease population size (but see [15]), and depressed population sizes can slow adaptive responses and directly lead to extinction via demographic stochasticity, mutational load, inbreeding, and Allee effects [16]. The rate of an adaptive response scales with both heritability (more specifically additive genetic variance [17]) and the strength of selection. The latter may either increase with maladaptation, leading to a stable degree of evolutionary lag [18], or decrease, potentially crossing a tipping point to extinction [14]. Beyond these interactions, other effects of environmental change such as direct induction of plastic changes [11,19–21] have also been explored.

Despite these areas of progress, little attention has been paid to feedbacks between maladaptation and another key driver of the rate of adaptation: generation time. Short generation time *per se* is recognized as a key ingredient in the success stories of evolutionary rescue—resistance to chemical attack in pest species like rabbits, mosquitoes or microbes—or a critical handicap explaining the dearth of examples of evolutionary rescue in long-lived vertebrates [22]. While the effect of generation time on the rate of evolution may be simple to predict, how generation time itself emerges from the complex interactions among rates of growth, birth, and death across ages or stages is less so. These interactions make it difficult to isolate the effects of generation time on evolutionary rescue from other, correlated factors like resilience to short-term environmental fluctuations [23]. For example, higher adult mortality rates can speed up the response to selection by increasing turnover [24–29], but may come with relevant downsides like a decrease in population size.

In complex stage-structured models of adaptation in specific populations [30], direct effects of shifts in generation time may be hard to disentangle from the suite of changes in other traits. At the other extreme, generalized models of evolutionary rescue often assume fixed generation times or model reproductive success with a single, composite fitness trait that combines mortality and fecundity [11,18,19,31–35]. To understand how generation time increases or decreases as a population faces a changing environment, we constructed a new model of evolving demographic traits that bridges simple, single-trait models and complex, stage-structured models. Our model predicts how generation time, the rate of adaptation and population fate (extinction or persistence) all vary as populations decline due to maladaptation. We find that generation time and its resulting impact on population persistence through adaptation depend on how both maladaptation and density dependence affect population vital rates. Using simulations, we show that the variable generation times emerging from our model determine disparate ranges of the rates of change that species can withstand via adaptation. We discuss these substantial disparities in light of life-history variation and novel threats to plant and animal species. Our model demonstrates that variation in generation time due to the interaction of species' life-history and environmental change is a quantitatively significant and previously overlooked driver of differences in the success of evolutionary rescue.

## 2. Model and results

Models of evolutionary rescue must explicitly combine ecology and evolution and are therefore diverse, including quantitative-genetic models in steadily changing environments [18], mutation-limited adaptation models with explicit loci and either sudden [4,36] or gradual [37] change, variant frameworks like Fisher's geometric model [38], and unifying frameworks that combine several approaches (e.g. [39]). We focus here on a model derived from quantitative genetics, in which standing variation among many unlinked loci, rather than the input of novel mutations of large effect, is the primary driver of adaptation.

Most models of evolutionary rescue restrict the effects of maladaptation to a single trait, such as absolute fitness or carrying capacity [34,35]. However, even the simplest organism has multiple demographic traits, such as birth, death, and growth rates, that could each be separately affected by environmental change. The most basic two-parameter model in continuous time is *r* = *b* − *d*, with the Malthusian parameter *r* defined as the difference of *per capita* birth and death rates. A few models have considered the effects of variation in both birth and death parameters in weak-selection [40] or rescue scenarios, with the latter focusing on demographic stochasticity in mutant lineages [41] or entire populations [42]. In particular, Klausmeier *et al*. [10] examined a set of models in which both density dependence and maladaptation could affect either recruitment or mortality rates. Here we rederive and generalize this framework, reaching new conclusions about how the interactions between demography and maladaptation determine the rate of adaptation.

Consider a population in which organisms are hermaphroditic, mature instantly and do not senesce, and reproduce sexually with overlapping generations. Given these assumptions, we ignore age structure, modelling the population via a single category of adults. Without variation in growth or maturation rates, generation time—the average age of mothers of new recruits to a population—varies with the rates of birth and death. Individuals are distinguished only by the value, *z*, of a single quantitative trait, which is set at birth and does not vary over an individual's life. The fitness of an individual is measured by *r*, the rate of increase in continuous time, which is the difference between recruitment and death processes. Although we model development as instantaneous, ‘recruitment' can be thought of as combining both birth rates and survival of juveniles to maturity.

We model negative density dependence as a decrease in recruitment and/or an increase in the *per capita* death rate with population size *N*. The parameter *ϕ*, which is defined to range from zero to one, controls the balance between these effects on each vital rate. When *ϕ* = 0, all density dependence occurs via decreased recruitment, and when *ϕ* = 1, all density dependence occurs via increased death rate. Additionally, the degree of maladaptation, which is defined as a mismatch between an organism's phenotype, *z*, and the optimum phenotype determined by the environment, *z*_opt_, similarly may reduce fitness by decreasing recruitment and/or increasing mortality. Let *x* represent the degree of maladaptation for an organism *i*, defined as *x_i_* = |*z*_opt_ − *z_i_*|. We parametrize the effects of maladaptation on vital rates with a parameter *β* which is defined similarly to *ϕ*: *β* = 0 indicates that the effects of maladaptation occur solely via reduced recruitment, and *β* = 1 indicates that maladaptation affects only death rate. Note that neither *φ* nor *β* modulate the strength of density dependence or maladaptation on demography.

Given this formulation, recruitment rates vary between zero and a maximum value which is realized in the absence of density dependence and maladaptation. Death rates have a minimum attained under those same conditions and can grow without bound as *N* and maladaptation increase. Without loss of generality, we set the maximum rate of recruitment to one and define the minimum rate of mortality relative to maximum recruitment as the parameter *d*_0_. We can then write fitness as the difference between a *per capita* recruitment (positive term in equation (2.1)) and death rate (negative term):

2.1 $$r(x,t)=d_{0}^{\phi }\left(\frac{h{\left(N\right)}^{1-\phi }}{\;f{\left(x\right)}^{1-\beta }}-\frac{d_{0}f{\left(x\right)}^{\beta }}{h{\left(N\right)}^{\phi }}\right).$$

Here, *f*(*x*) represents the penalty caused by maladaptation in trait *z*, and can be any monotonically increasing function for which *f*(0) = 1 and lim*_x_*_→∞_ *f*(*x*) = ∞. Similarly, *h*(*N*) represents density dependence and can be any monotonically decreasing function of population size *N* with the constraints *h*(0) = 1 and lim*_N_*_→∞_ *h*(*N*) = 0. The factor $d_{0}^{\phi }$ scales overall rates such that generation time is equal to $d_{0}^{-1}$ for a perfectly adapted population (*x* = 0) regardless of the values of *β* and *ϕ*. Also, if we assume that changes in *N* and *x* are slow compared to an individual's lifespan, we can approximate lifetime reproductive success as the birth rate multiplied by the mean lifetime, which is the reciprocal of the death rate. Applying this to equation (2.1), we see that all terms with *β* or *ϕ* cancel, yielding $W_{i}(x)\approx h(N)/(f(x)d_{0})$. This indicates that *β* and *ϕ* have little direct effect on reproductive success unless selection or demography are changing rapidly relative to lifespans.

Equation (2.1) has some similarities but also key differences when compared to the closest precedent [10]. The most important difference is that here, maladaptation has a multiplicative effect on each trait, reducing it in proportion to its magnitude rather than entering the expression via subtraction. We believe that this functional relationship is the most appropriate because, while *r* is a net rate of change and can be positive or negative, its constitutive terms are each the rates of Poisson processes—namely, recruitment and mortality—and cannot be negative. Explicitly considering how *b* and *d* comprise *r* bounds the reasonable range of values for *r*. For example, if maladaptation drives birth rate to zero without raising mortality rates, then *r* should be no lower than −*d* (as noted in [14]); our formulation naturally incorporates this constraint, while additive versions do not.

If we make the simplifying assumption that the population is monomorphic and that *z*_opt_ is constant over an organism's lifetime, we can solve for population size at equilibrium $\hat{N}\;$ by setting the right-hand side of equation (2.1) to zero, which produces a simple, general relation $h(\hat{N})=d_{0}f(x)$. Given the constraints on these functions listed above, an equilibrium exists for any reasonable minimum rate of death, 0 < *d*_0_ < 1. Assuming that the population is near demographic equilibrium and that all individuals have similar vital rates, we approximate generation time *T* as the inverse of the recruitment rate (see electronic supplementary material, appendix, for details). This yields a simple expression for $\hat{T}$, the generation time at equilibrium:

2.2 $$\hat{T}=\frac{\;f{\left(x\right)}^{1-\beta }}{d_{0}^{\phi }h{\left(N\right)}^{1-\phi }}.$$

Substituting for $h(\hat{N})$ and simplifying, we obtain

2.3 $$\hat{T}=\frac{\;f{\left(x\right)}^{\phi -\beta }}{d_{0}}.$$

Therefore, our model predicts that at a fixed level of maladaptation, generation time at equilibrium will increase exponentially with the difference between *ϕ* and *β*. To quantify these predictions, specific choices must be made for *f*(*x*) and *h*(*N*). For density dependence, we focus here on the logistic function, modified to suit a birth–death framework, *h*(*N*) = 1 − *N*/*M*. Here, we set a desired equilibrium population size *K*, then set the maximum population size *M* = *K*/(1 − *d*_0_) to ensure that recruitment balances deaths when *N* = *K*. In the main text, we focus on *f*(*x*) = exp(*x*/*σ_s_*). The parameter *σ*_s_ is set such that the population attains an equilibrium size of one-tenth of *M* when *x* = 5. While arbitrary, this choice standardizes the strength of selection across different functions.

**Figure 1.**
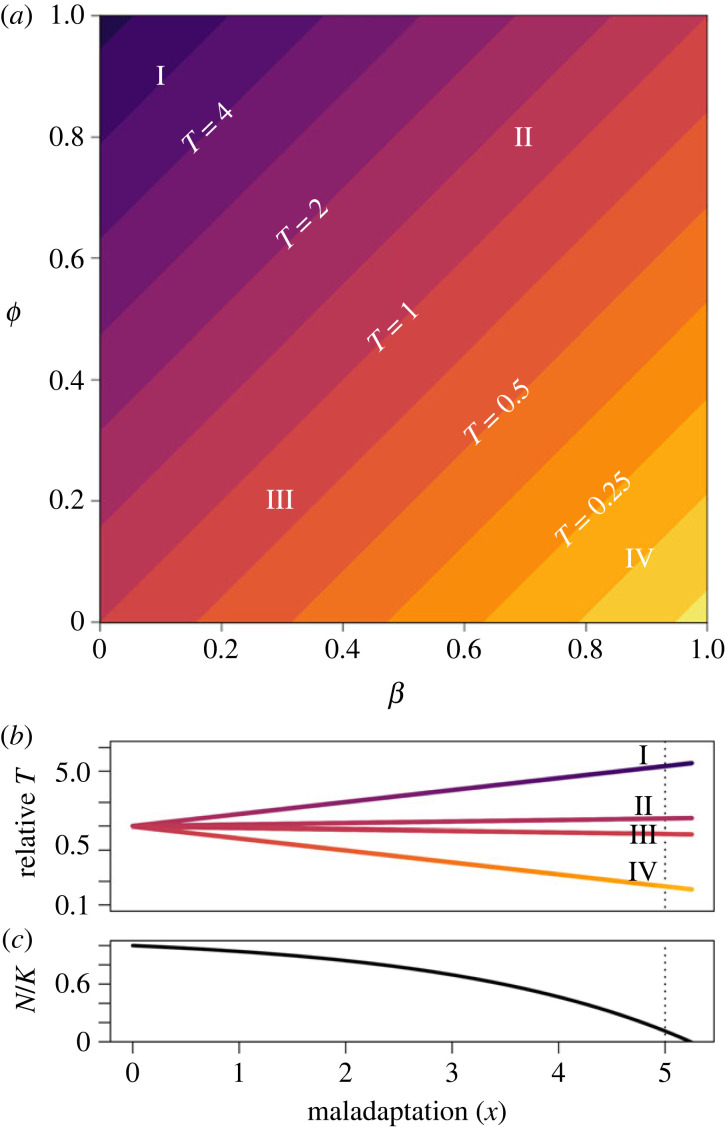
Predicted equilibrium generation times, $\hat{T}$, relative to $d_{0}^{-1},$vary according to the difference between *ϕ* and *β*. (*a*) Generation time given a phenotypic mismatch *x* = 5 (equation (2.3)). Roman numerals represent parameter combinations depicted in (*b*). (*b*) Responses of relative generation times to variation across a range of values of the degree of maladaptation *x*. (*c*) Equilibrium population size, as a ratio *N*/*K*. Note that equilibrium population size declines with *x* but is not perturbed by *β* or *ϕ*.

As depicted in figure 1, generation time will become longer with increased maladaptation (slowing adaptation) when the environmental mismatch affects recruitment to a greater extent than it does density-dependent competition (scenario I: low values of *β*, high values of *ϕ*). Conversely, generation time will become shorter with increased maladaptation (speeding adaptation) when the environmental mismatch increases adult mortality to a greater extent than does density-dependent competition (scenario IV: high values of *β*, low values of *ϕ*). Also, note that the equilibrium population size is not predicted to be influenced by *β* or *ϕ* (figure 1*c*).

**Figure 2.**
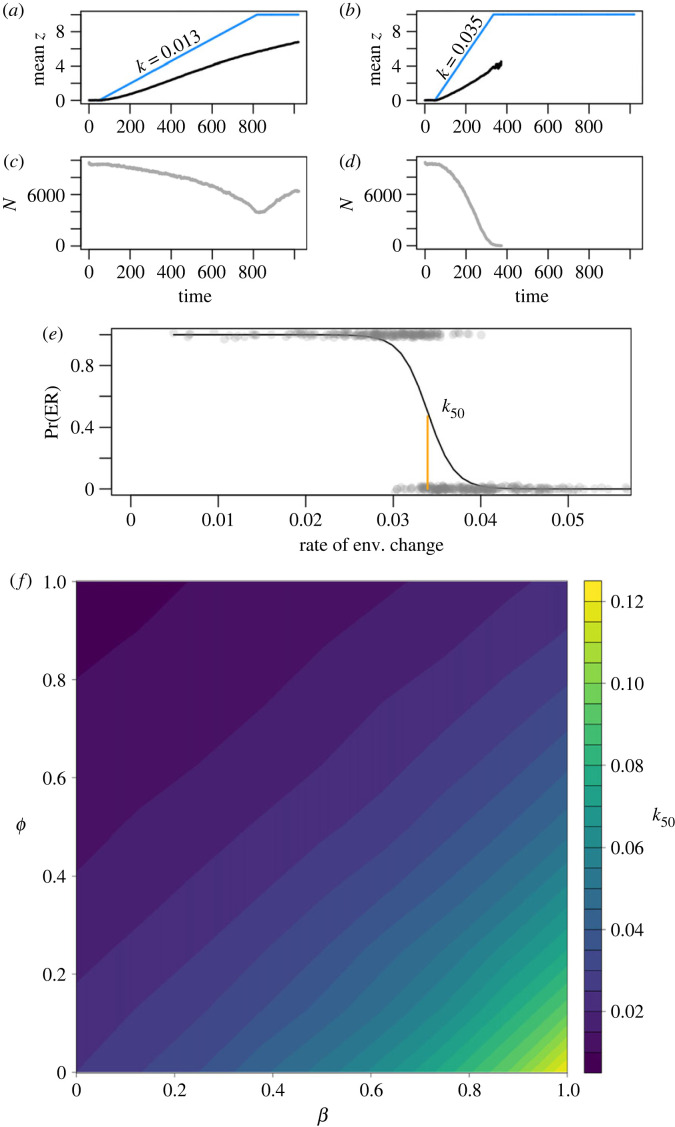
Evolutionary rescue simulations across a range of *ϕ* and *β*. (*a,b*) Examples of mean (black) and optimal phenotype (*z*_opt_; blue) and population sizes (grey; (*c,d*)) for a persisting population (*a,c*) and one that goes extinct (*b,d*); *k* indicates the rate of change per time unit. *d*_0_ is 0.1, producing a base generation time of 10 units. Example logistic regression (*e*) of 200 replicates across a range of rates; *k*_50_, the rate for which 50% of populations are estimated via logistic regression to survive via evolutionary rescue, is determined as the inflection point of the fitted curve. (*f*) *k*_50_ values across a range of *ϕ* and *β*. A minimum of 125 observations of both extinctions and rescues were performed for each of 81 parameter combinations. Fitness is determined by *f*(*x*) = exp(*x*/2.28); genomic mutation rate *U* = 0.01.

To understand how well our predictions could capture qualitative dynamics in complex, non-equilibrium scenarios, we simulated rescue experiments in large (*K* = 10 000) polymorphic populations in which demographic rates are governed by equation (2.1). We first generated populations with many (1000) loci, each with two possible alleles with small, Gaussian-distributed effect sizes (*σ* = 0.035), and set allele frequencies to yield target values of additive genetic variance, *V*_A_ (see electronic supplementary material, computational methods). Populations were sexual with no linkage and reproduced in continuous time (i.e. overlapping generations) with selection acting on fecundity and mortality in accordance with the parameter *β*. Mutations could change one allele to the other; the model did not permit the addition of novel alleles, beyond the two specified per locus. We then challenged the population to adapt to environmental change by increasing the optimal phenotype *z*_opt_ at a rate *k* to a maximum change of 10 units (figure 2*a*,*b*). The size of this perturbation required substantial adaptation to allow persistence, but was well within the envelope of phenotypic variation allowed by standing genetic variation. The rate *k* was varied across replicate simulations according to a simple optimization routine designed to estimate the rate, designated *k*_50_, at which a population was equally likely to persist or go extinct (figure 2*c*–*e*). These values varied over more than an order of magnitude with *ϕ* and *β* (figure 2*f*), qualitatively matching the variation predicted by equation (2.3). Owing to substantial standing genetic variance, the role of mutation during the period of rescue was very small (electronic supplementary material, figure S1). *k*_50_ was found to vary proportionally to initial *V*_A_ (electronic supplementary material, figure S2), and very similar dynamics were observed for an alternative fitness function *f*(*x*) = 1 + *x*^2^/*σ_s_* (electronic supplementary material, figure S3). Qualitatively identical results were also obtained when *V*_A_ was allowed to accumulate to mutation–selection–drift balance (electronic supplementary material, figure S4).

**Figure 3.**
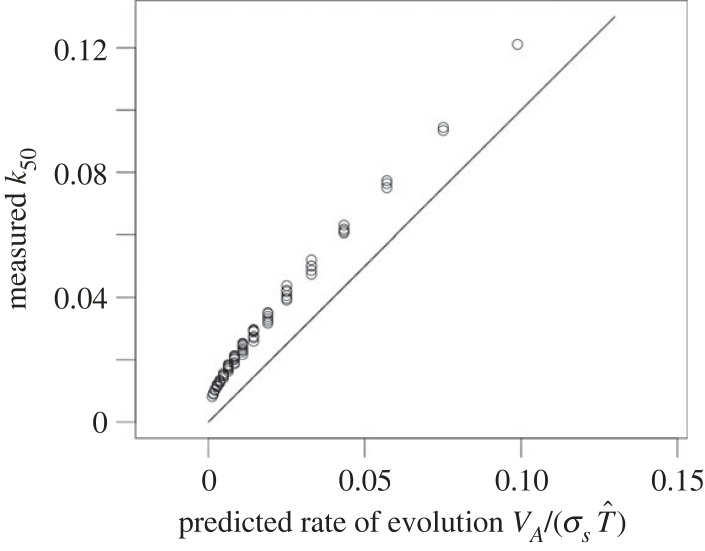
Predicted rates of evolution $V_{A}/(\sigma_{s}\hat{T})$ (*x*-axis) compared to measured *k*_50_ values from the 81 combinations of *ϕ* and *β* underlying figure 2*f*. Predictions are calculated at a high level of maladaptation (*x* = 5). Line indicates *x* = *y*. Simulation data and parameters are those in figure 2.

Given the complexity of the eco-evolutionary dynamics in these simulations (e.g. figure 2*a*,*c*), we sought to clarify the role of generation time in population persistence. To quantify the correspondence between the patterns in figures 1 and 2, we estimated the rate of evolution for maladapted populations by multiplying the initial *V*_A_ by the fitness gradient for a monomorphic, equilibrium population, $\sigma_{s}^{-1}$ and dividing by $\hat{T}$. Figure 3 shows these results for *x* = 5, corresponding to equilibrium population sizes of one-tenth of the maximum, *M*. These rough estimates, which ignore phenotypic variation and non-equilibrium dynamics, nonetheless display a very high rank-order correlation with *k*_50_ (*R*^2^ = 0.997) regardless of the chosen value of *x*. This very strong relationship suggests that predicted variation in $\hat{T}$ explains the vast majority of variation in capacity for evolutionary rescue in our simulations.

Next, we simulated an instantaneous environmental change to a new value of *z*_opt_ that was then maintained until the population went extinct or met persistence criteria (see electronic supplementary material, computational methods). Neither *ϕ* nor *β* had any significant effect on the maximum tolerable change in this scenario (electronic supplementary material, figure S5), consistent with their effects being mediated by their impact on generation time alone. Additionally, for populations evolving in an environment changing at their estimated *k*_50_ (for which 50% are expected to adapt and persist) we measured the cumulative number of generations during the period of change. Whether generation time was measured by counting births or deaths, we found that realized mean generation times in persisting populations varied in qualitative agreement with figure 1 and equation (2.3) (electronic supplementary material, figure S6). Together, these lines of evidence show that generation time varies substantially across the parameter space of our model and has a predictable impact on the process of evolutionary rescue.

**Figure 4.**
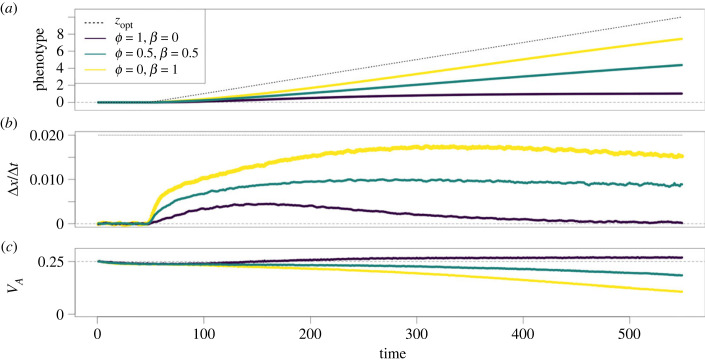
Ensemble means of mean phenotype (*a*), change in mean phenotype per time unit (*b*), and additive genetic variance (*c*) for three values of *ϕ* and *β*. The rate of environmental change, depicted by the dotted line (*a*,*b*), is *k* = 0.02. Additive genetic variance is measured as the slope of midparent–offspring regression. Changes in phenotypes are smoothed with a simple uniform moving average of five units. At least 300 replicates are averaged for each condition.

For a given *k*, figure 4 illustrates how *ϕ* and *β* determine the dynamics of adaptation. While previous analyses of moving optima focus on equilibration of the degree of maladaptation *x*, in our simulations *x* does not settle to a steady value. One contributor to this non-equilibrium behaviour is the loss of additive genetic variance, which is particularly acute in populations with larger adaptive responses (figure 4*c*). While simulations were designed with a large number of loci with small allelic effects specifically to stabilize additive genetic variance, the assumption of constant *V*_A_ made in previous analyses [31] breaks down in our model under intense, prolonged selection. In a variant of our model with artificially stable *V*_A_, populations could achieve steady values of *x* (electronic supplementary material, figure S7); variation in *k*_50_ with *ϕ* and *β* in this model was very similar to that seen in figure 2 (electronic supplementary material, figure S8).

**Figure 5.**
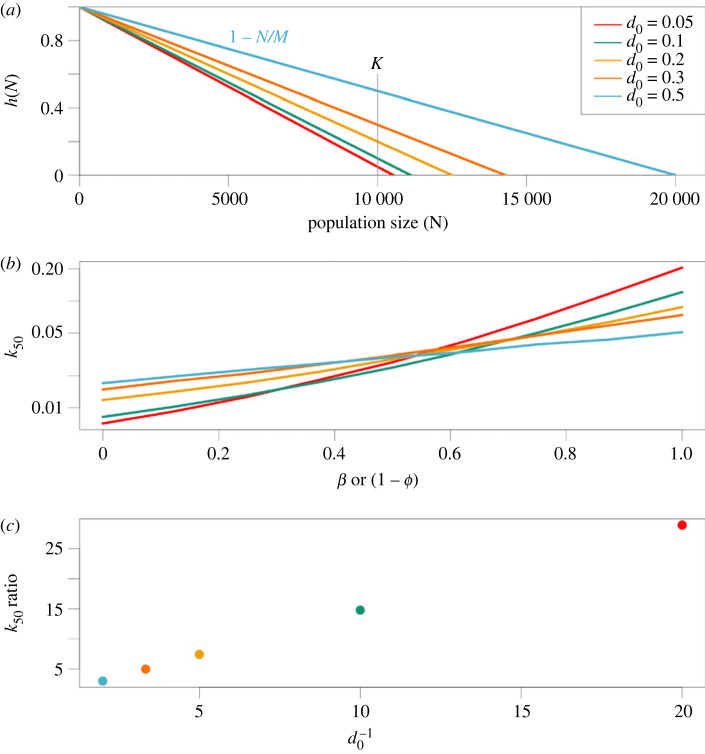
Capacity for demographic compensation determines capacity for evolutionary rescue. (*a*) The *x*-intercept denotes the parameter *M* = *K*(1 − *d*_0_), which is used to scale the relationship between density-dependent population growth and *d*_0_. This scaling ensures that the equilibrium population size is equal to *K* across values of *d*_0_. Higher values of *h*(*N*) indicate less restriction by density dependence. (*b*) Estimated rate of environmental change at which 50% of populations survive (*k*_50_) based on simulations for parameter combinations satisfying *ϕ* + *β* = 1. This equation is the diagonal from slowest to fastest generation times in figure 1 and captures the main axis of variation in *k*_50_ in figure 2*f*. (*c*) The ratio of *k*_50_ (*ϕ* = 0, *β* = 1) : *k*_50_ (*ϕ* = 1, *β* = 0) as estimated from simulated data in (*b*), plotted against the reciprocal of *d*_0_. Note that (*b*) is on a log–linear scale, making the ratio of the highest to lowest values an appropriate measure of the slope.

Variation in generation time depends on demographic compensation—decreases in density-dependent mortality or increases in density-dependent births that occur with declines in population size [43]. The parameter *d*_0_, which represents the ratio of the density-independent death rate to the birth rate as *N* → 0, determines the compensatory capacity in our model. Figure 5*a* illustrates how we construct models with varying values of *d*_0_ while maintaining the same equilibrium population size *K*. As *d*_0_ increases, there is a corresponding decrease in the slope of *h*(*N*), which dictates the contribution of density dependence to population growth. To evaluate the effect of this demographic shift on evolutionary rescue, *k*_50_ was estimated for various values of *d*_0_ for parameters *ϕ* + *β* = 1 (the *y* = 1 − *x* diagonal in figures 1*a* and 2*f*). The effects of *ϕ* and *β* on evolutionary rescue, both positive and negative, are attenuated with increasing *d*_0_ (figure 5*b*). The ratio of *k*_50_ for the most (*ϕ* = 0, *β* = 1) and least (*ϕ* = 1, *β* = 0) favourable parameters is plotted as the *k*_50_ ratio in figure 5*c*; this measure of sensitivity correlates very strongly with $d_{0}^{-1}$, or the ratio of birth to death rates when *N* = 0.

## 3. Discussion

While a population that is declining toward extinction is clearly unfit, the abstraction of evolutionary fitness, measured as lifetime reproductive success, is not sufficient to predict evolutionary rescue in complex situations. By considering the minimal additional complexity of distinct recruitment and death rates, we have shown that rescue depends on ecological feedbacks that change generation time, even without considering other factors like age at maturity and somatic growth. These changes are governed by both the biological impact of the environmental change but also the pre-existing mechanisms regulating density in the population, revealing generation time as an emergent property of both a species and its environment. Generation time has been sometimes neglected in lists of organismal characteristics predicting evolutionary rescue (e.g. [6,33]), likely because its role seems obvious and uncomplicated: shorter generation times yield faster rates of evolution. Perhaps consequently, many models of evolutionary rescue measure time exclusively in generations [1], missing differences between species in the potential for evolutionary rescue highlighted here.

Emergent changes in generation time are driven by compensation in vital rates as populations decline. These compensatory responses have been related to the continuum of slow to fast life histories [44], as populations with shorter generations turn over faster, and are better able to compensate for anthropogenic mortality [45]. Recent work continues to define these complex connections between density-dependent regulation of populations and the evolution of life histories on this continuum [46]. Variation in regulatory processes can be orthogonal to variation in generation time if density-dependent mechanisms affect juveniles and adults separately, as in species that are long-lived but have very high fecundity such as trees or temperate benthic fishes [47,48]. Our approach here recognizes that population demography and generation time emerge from the combination of density-independent processes governing baseline mortality and recruitment rates at low density (*d*_0_) and explicit mechanisms of negative density dependence (*ϕ*) for a given population. By focusing on generation time, our results can be related to variation on the slow–fast life-history spectrum, but also consider density-dependent processes modulated by *ϕ*, in an orthogonal direction.

Demographic responses to a sudden jump in the environment can speed or slow generation time, but as modelled here, these changes would only determine how quickly extinction may occur, not the likelihood of rescue. However, many relevant threats are gradual. For example, slow and steady changes in phenology of plants and insects have led to reduced recruitment of juveniles in great tits, but is compensated by higher juvenile survival [49]. In our model, this would correspond to low values of both *ϕ* and *β*, suggesting little change in generation time with maladaptation. Our model results imply that the situation is most dire for long-lived species in which anthropogenic threats primarily affect processes determining the recruitment rates of juveniles. Novel threats to fecundity and juvenile survival, if compensated by lower adult mortality rather than increased recruitment due to lower density, can drive increases in generation time, slowing evolutionary responses and pushing population trajectories into a downward spiral. Aspects of this process can be observed in species for which recruitment has repeatedly failed, leading to a geriatric age structure. For example, degraded environmental conditions have led to chronic recruitment failures in eastern hellbender populations, due to both reduced availability of suitable nest cavities and increased rates of filial cannibalism by males [50]. Given enough time, current conditions could favour non-cannibalistic males, leading to the loss of the cannibalistic behaviour, but increased generation time could impede the pace of evolution out of this trap. By contrast, emergent pathogens that induce mortality in adults can sometimes induce demographic compensation in recruitment [51], potentially driving changes in generation time advantageous for rescue. Pond-breeding amphibians threatened by chytrid fungus may be an example of a novel threat mostly concentrated on adult survival [52] in populations that are strongly limited by competition among juveniles [53]. For example, adult mortality caused by the fungus *Bd* was found to be compensated by greater recruitment in the boreal toad [54] and alpine tree frog [52]; our model predicts that generation time would accelerate in these systems, potentially driving more rapid rescue. Shortened generation times due to demographic responses are also indicated in badger populations threatened by bovine tuberculosis that was compensated by greater recruitment [55], and in Tasmanian devils suffering the transmissible devil facial tumour disease [56].

Compensation and changes in generation time are also relevant for understanding eco-evolutionary dynamics of populations in which adults are harvested, including managed forests, fisheries and wildlife subject to hunting or culling. Management practices and other sources of adult mortality can shape generation time and determine adaptive response in trees [24,57]. Adaptation to climate change, which can increase mortality of trees via the combination of higher temperatures and more frequent droughts [58], could accelerate in circumstances in which recruitment can at least partly compensate for adult mortality, though such compensation is not always observed [59]. Analysis of long-term fisheries datasets has shown that the removal of adults in cod fisheries has led to the evolution of earlier maturation [60,61]. When fishing mortality also includes smaller individuals, reduced competition can cause compensatory somatic growth, allowing juveniles to reach reproductive size thresholds earlier in life [62]. Evolutionary and ecological responses appear to have synergistic effects on decreasing generation time, which may have allowed cod populations to adapt to sustain intense levels of exploitation for generations (although many stocks eventually collapsed due to overfishing). While faster maturation is a direct adaptive response to size-selective fishing pressure, it may, through its effects on generation time, serve to speed adaptive responses to other threats such as climate change.

The responses of wildlife to hunting mortality illustrate that density-dependent regulation limits populations in complex ways, and reveal how additional mortality may or may not lead to a compensatory demographic response and change in generation time. Experimental harvest of willow ptarmigan showed that moderate levels of hunting mortality was compensated for by a decrease in natural mortality, primarily from gyrfalcons [63] which show both numeric and functional responses in attack frequency according to ptarmigan density [64]. Social and behavioural interactions also mediate potential changes in generation time. For example, culling of adult jackals disrupted the population's social structure, leading to earlier maturation times and dispersal into the managed area by young from source populations [65]. Hunting of adults similarly disrupted the structure of breeding groups in pumas, but in this case, frequent male turnover and increased rates of infanticide, combined with declines in female survival, led to overall population declines, and changes in generation time have not yet been observed [66].

Building on the foundation established here, future work could productively add complexity to either the evolutionary or demographic components of our model. Assuming constant genetic variation may overestimate the probability of rescue when populations experience significant declines and/or adaptive responses [13,67,68], as illustrated in our simulations. Future work could examine how genetic architecture and demography influence this loss to enable more fine-grained predictions of long-term adaptive tracking. Demographic processes are also important for the fate of new variants arising from mutations or rare combinations of alleles produced by recombination. These considerations may also affect the rate of adaptation, particularly when linkage is present. The simple demography considered here could be expanded to include age structure, maturation time, somatic growth rates and senescence. Future work could consider evolving demography in a community context, as interactions with predators, prey and competitors could readily produce strong selection pressures on specific life stages. For example, both Jones *et al*. [69] and Osmond *et al*. [27] find that predation can, in some circumstances, accelerate the adaptation of prey to a changing environment. Novel threats can induce plasticity in vital rates beyond their effects on density and directly select for changes in life histories [51]. Future work could model the interactions of demographic compensation, as explored here, with these other influences on generation time. The principles determining the potential for evolutionary rescue discussed here are relevant to the evolution of resistance to treatments in infectious disease and cancer [6]. In fact, the idea that maladaptation via lower birth rates versus higher death rates may provoke different adaptive responses has been noted in these fields, though typically with a focus on mutational input per unit time [23,41]. For example, Igler *et al*. [70] model the evolution of resistance to bacteriocidal versus bacteriostatic antibiotics, noting that bacteriocidal antibiotics can lead to greater mutational input under some conditions. Czuppon *et al*. [71] also find a difference in the probability of rescue across these antibiotic classes, which they analyse in the context of different modes of bacterial density dependence. Similarly, Alexander *et al*. [72] found differences in the probability of evolved resistance between drugs that prevent cells from becoming infected and those that reduce viral production in infected cells. In our simulations, high standing genetic variation greatly reduces the role of mutation, helping to tease apart the impact of generation time *per se* from mutational input. Future work could apply the insights found here to cases of mutation-limited adaptation characteristic of evolution in pathogens and cancer.

In summary, addressing existential threats to species requires detailed understanding of the multidimensional effects of demography on both the dynamics and adaptive potential of populations. Prior models of evolutionary rescue achieved simplicity by separating the fitness effects of alleles from the demographic processes of their population. Our results show this separation must be bridged to understand the evolutionary potential of populations subject to serious perturbations that change densities, age structures and threaten the persistence of species.

## Data Availability

The data are provided in electronic supplementary material [73].
